# Pioneering Construction Materials through Prototypological Research

**DOI:** 10.3390/biomimetics4030056

**Published:** 2019-08-13

**Authors:** Felix Heisel, Dirk E. Hebel

**Affiliations:** Sustainable Construction, Faculty of Architecture, KIT Karlsruhe, 76131 Karlsruhe, Germany

**Keywords:** prototypology, prototype, typology, architectural, research, methodology, mycelium, MycoTree, sustainable, construction

## Abstract

The article at hand follows the understanding that future cities cannot be built the same way as existing ones, inducing a radical paradigm shift in how we produce and use materials for the construction of our habitat in the 21st century. In search of a methodology for an integrated, holistic, and interdisciplinary development of such new materials and construction technologies, the chair of Sustainable Construction at KIT Karlsruhe proposes the concept of “prototypological” research. Coined through joining the terms “prototype” and “typology”, prototypology represents a full-scale application, that is an experiment and proof in itself to effectively and holistically discover all connected aspects and address unknowns of a specific question, yet at the same time is part of a bigger and systematic test series of such different typologies with similar characteristics, yet varying parameters. The second part of the article applies this method to the research on mycelium-bound building materials, and specifically to the four prototypologies *MycoTree*, *UMAR, Rumah Tambah*, and *Futurium*. The conclusion aims to place the results into the bigger research context, calling for a new type of architectural research.

## 1. Alternative Construction Materials

The construction industry today represents one of the biggest sinks of energy and raw materials, accounting for an estimated 32% of global energy consumption, 25% of CO_2_ emissions, 12% of water use, 40% of waste generated (by mass), and 40% of materials used [1,2]. The chair of Sustainable Construction at the Karlsruhe Institute of Technology (KIT) Germany and the Future Cities Laboratory (FCL) Singapore thus concentrates its research on alternative materials and their building application in specific contextual settings by taking into account the metabolism of material resources, local human capacities, and accessible skills and technologies. The alternative aspect of this focus emerges from an exploration of innovative and entrepreneurial thinking. The research follows two strong convictions: The future city cannot be built within today’s wasteful linear economic system but requires new alternative materials and circular construction technics; and the 21st century will face a radical paradigm shift in how we produce materials for the construction of our habitat [3,4]. It consequently calls for an adequate research methodology and/or method combining the many interdisciplinary aspects concerned, as well as spanning the bridge from basic research to application.

## 2. Approaching Architectural Research

Commissioned by the Royal Institute of British Architects (RIBA) Research Committee in 2007, architect, writer and educator Jeremy Till began his canonical paper on architectural research with the words: “There is still, amazingly, debate as to what constitutes research in architecture” [5]. Today, the debate has become somewhat clearer but certainly not less complex. Thus, when writing about methodologies in regard to our research on alternative construction materials, we want to position ourselves first in regard to research in architecture.

In his paper, Till continued by abandoning three myths, the third of these being: “Building a building is research”. Certainly, from the very beginning of architecture, research has been conducted through the process of building. Trial and error in design and construction, in combination with observation and study of the built environment and connected disciplines, often led and still leads to the application of new innovations. Consequently, in architecture, design and research have been understood as something closely (inter-)connected for many centuries, “neither polar opposites nor equivalent domains of activity. Rather, the relationship between the two is far more nuanced, complementary, and robust” [6]. According to Till’s argument, the flaw lies in the definition of the goal: “Architectural knowledge may lie in the building, but it also lies elsewhere: in the processes that leads to the building, in the representation of the building, in its use, in the theories beyond the building, in the multiple interpretations of the building and so on. Architecture exceeds the building as object, just as art exceeds the painting as object. Architectural research must, therefore, address this expanded field” [5].

Addressing the same (still existing) myth, in October 2017, architect Winy Maas, co-founder of the Dutch architectural firm MVRDV, encouraged the discipline through his book *Copy Paste* to stop suffering from its “syndrome surrounding originality”. The interesting part in this reasoning is that it understands the single building as part of a continuous architectural research (and not as research itself), following the definition of “science, which progresses via researchers building on the work of others” [7]. Speaking for the architectural practice, he continues: “Why not deepen our architectural analyses? Why not be open and honest about the references we make? Why not improve on the explorations, innovations, and suggestions of our predecessors?”

In one of the earliest compendiums on architectural research, author James Snyder already in 1984 had provided a commonly accepted definition of research in general; it is a “systematic inquiry directed toward the creation of knowledge’” [8]. Important in this context is the term ‘systematic’: Firstly, it further strengthens Till’s argument that a building by itself may not count as research, since design decisions in the architectural office are—even though often research-based—usually not systematic. Secondly, in the academic world, systematic directly implies the need for a research methodology, which can be understood as a constructive framework to structure and describe the utilized research methods.

At KIT and FCL, we understand our architectural research through prototypological methods as an extension of these arguments: While Till and Maas already understand the building as only one part within an extended and successive research timeline, they describe it as a single and self-contained entity. This article, however, advocates a shift in focus from the singularity of the object towards the act of building as a method, and with it, a shift in focus from a formal manifestation towards an adaptable configuration based on a multitude of separate yet interconnected research results in the fields of material research and construction technologies, addressing Till’s expanded field of architectural research through a systematic methodology.

## 3. Through Prototypological Research

While the most common research methods are qualitative, quantitative, conceptual, applied, analytical, descriptive, empirical or fundamental, within (academic) architectural research, and within the mindset of a paradigm shift in the construction industry described above, we feel that our method can be best described as “prototypological”. Joined from the terms “prototype” and “typology”, prototypology represents a full-scale application, that is an experiment and proof in itself to effectively and holistically discover all connected aspects and address unknowns of a specific question, yet at the same time is part of a bigger and systematic test series of such different typologies with similar characteristics, yet varying parameters. The term prototypology was defined by architecture historian and publisher Andreas Ruby in 2003 in the Metapolis Dictionary of Advanced Architecture as: “Just as the prototype anticipates a product yet to be developed, the proto-typology represents a typological configuration in permanent state of evolution. Whereas a conventional typology defines a generic model of organisation, which becomes specific through its application, the prototypology is specific from the beginning. On the other hand, it never really becomes generic as it continues to transform itself through the information it receives. As a pliable, learning matter, it adapts to the changing needs of programs and users. Hence, a prototypology is not a model, but a transient phase of an evolutionary process and, therefore, always ahead of its type” [9]. Building on Ruby’s definition, consequently, the goal of such a prototypology cannot be a formalistic debate but rather the discovery and verification of skills, information, culture, climate, material, techniques, and other soft and hard parameters within the given context. By absorbing and reacting to its surrounding information and matter, prototypology is always specific and represents the counterpart to a prototype that—designed and approved as generic solution—is replicated and applied without knowledge of or sensitivity for the situation it might find itself in.

The modernist era was obsessed with the idea that architectural objects could be designed and applied the same way as industrially produced serial products. Jean Prouvé’s Maison Tropical is a good example. Planned for an application on the African continent, Prouvé developed a housing typology which was able to accommodate the extreme climatic conditions of the tropical context. However, designed with state-of-the-art sun-protection and ventilation systems, and built in aluminum for weight reasons (as it was planned to be constructed in France and air-dropped into various areas in Africa), the initial typology turned into a (proto)type that did not allow for any changes or adaptations. It was designed just like a car, as a serial product to be deployed into any context and used as designed.

Prouvé’s prototype—in total, three were built and tested in Niger and Congo in 1949 and 1951—embodies failures of the modernist movement in two distinct points: first, understanding architectural design as the act of creating a single prototype to be replicated independent of the multilayered contextual parameters important to the discipline; and second, a misunderstanding of foreign and development aid campaigns: Constructed as a closed entity that cannot be informed once deployed in a specific context, the prototype will always stay a stranger unable to integrate into its new context. Providing prototypes creates a dependency that might be helpful in the short term but fatal in the long run, as it disables self-steered and self-informed adaptation processes and development.

Therefore, we believe that especially architectural research needs to be seen as open and adaptable. In today’s academic architectural research, we therefore welcome the increasing acceptance and importance of architectural demonstrators, pavilions, and physical experiments. However, in terms of defining such full-scale applications, it is important to clarify their role within the overall research agenda: While a demonstrator often provides the proof for an assumption, the prototype is understood as preparation for the serial production of a final product. Prototypology’s justification, however, is the process of its creation, and it can thus represent all kinds of typological configurations in a permanent state of evolution. This does not always require a full-scale building. The development of a prototypology can be understood as a constant testing and adaptation process of relevant aspects of architectural practice. The method of a prototypological development therefore also allows the (temporary) neglect of certain aspects of building in favour of an in-depth concentration on other specific research fields—for example, tectonic, constructive or structural–physical qualities. Therefore, within the research methodology, prototypologies can be dispersed in various stages of the process development and even applied with changing objectives in different ‘hosts’, as illustrated in Section 4. The method we propose allows for a dispersed understanding of the architectural body in order to be informed and reintroduce knowledge into a continuous development loop of adaptation.

Looking at the timelines of our own research agendas, the method of the prototypology seems to be very effective rather early on in the process. It represents an element we refer to as “fast-forward”, a quick look into the future by concentrating on certain aspects and possibly neglecting others. Prototypology, thus, aims to be a sort of pioneer, producing a variety of results based on early tests or even hypotheses, across a wide array of fields and parameters of possible future applications. The experimental character of such a structure embodies the group’s interest in new discoveries, and in the successful (or unsuccessful) crossing into new territories of the act of building, rather than an affirmative proof of something already known.

**Figure 1:**
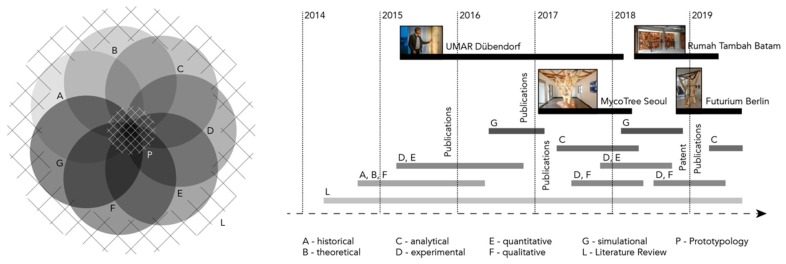
The conceptual framework and timeline for the mycelium-bound building elements research at KIT Karlsruhe and FCL Singapore, positioning the prototypologies in regard to time and context of the overall research agenda and their applications. Drawing by Felix Heisel (2019), in parts based on [6].

Prototypology is consequently one method within a multidimensional research methodology (see Figure 1) including and combining various other (admittedly often tedious) methodical inquiries and iteration processes, leading in parallel also to commonly known communication channels, such as scientific publications, papers or journal articles that will mostly address very specific aspects of the development as discussed before. The ultimate state of the described research is therefore not a single building or a single self-contained academic piece of work but rather the accumulative overlay of adaptations in a continuous process. Embedded into the conceptual framework of the group, the method derives and validates its original research questions from an extensive literature review and the resulting assessment and critique of present perspectives.

Last, but not least, prototypology also acts as an eye catcher for fellow scientists and the interested community—often leading to collaborations and/or funding options, which would have been unthinkable without their construction. As such, it takes the creation and explicitly the communication of knowledge from Snyder’s research definition very seriously—as it allows and encourages other researchers to build on and critique the publicly shown results in any of the available methods of Figure 1. Unquestionably, prototypology also simply represents an architectural application (full or partial as discussed before), probably the easiest and most successful tool the discipline has at hand to discuss research questions and measure their effects, certainly when compared with drawings, visualizations or text, for example.

While the construction of an architectural experiment in the scale of Till or Maas often involves the input of a practicing architect and engineer—as it might need to adapt to given legal frameworks—the object is always embedded in a long-term, systematic investigation and is, thus, part of academic architectural research as discussed by Snyder. Being able to combine these two elements and possibly bridge a rather artificial divide of building and research, we believe that prototypology provides an effective method to address and facilitate the paradigm shift on how we construct our cities of the future. The university setting, and prototypology’s function, certainly allows for such an approach due to the freedom of choosing its own determining parameters before applying them in a more complex and holistic context. Therefore, we agree with Till, questioning the assumption that “building a building is research” and propose to extend Till’s call to place a single building into an expanded field of knowledge, by shifting the expanded field into the act of building. In this sense, an architectural research project has many sites and many clients as well as many settings, restrictions, possibilities, and responsibilities—in short, many prototypologies. As Till speaks of the building as an object, we would like to speak of architectural prototypologies in their superimposition as a new expanded architectural body.

## 4. Mycelium-Bound Building Elements—Architectural Research as a Superimposition of Prototypologies

In order to illustrate the concept of a prototypological architectural research, this chapter describes an ongoing research project on mushroom mycelium-bound building elements as a future source for the construction industry. As shown earlier in the timeline of Figure 1, this research stream began in early 2014 with the first (literature) studies on the properties and capabilities of mycelium, the vegetative part of mushrooms. This dense and fast-growing matrix of single hyphae can act as natural glue. While digesting plant-based waste products such as sawdust, mycelium binds this substrate into a material composite with controllable properties. The common advantages of such products are significant: As mycelium follows a metabolic cycle, building elements or whole constructions can be composted after their use. Materials can be grown locally, reducing both energy and time required for transportation. Further, as they are organic matter, they act to reverse carbon emissions through absorption. While those facts are common to mycelium research at large, climate conditions and therefore growth conditions, substrate quality and composition or mushroom strand selection are specific parameters that define varieties and create the diverse field of possibilities within the project and its applications. Mushroom-bound materials can therefore be anything from a sponge-like insulation or acoustic panel to dense and structural boards for construction, which in total form the expanded field or the dispersed body of the research.

**Figure 2:**
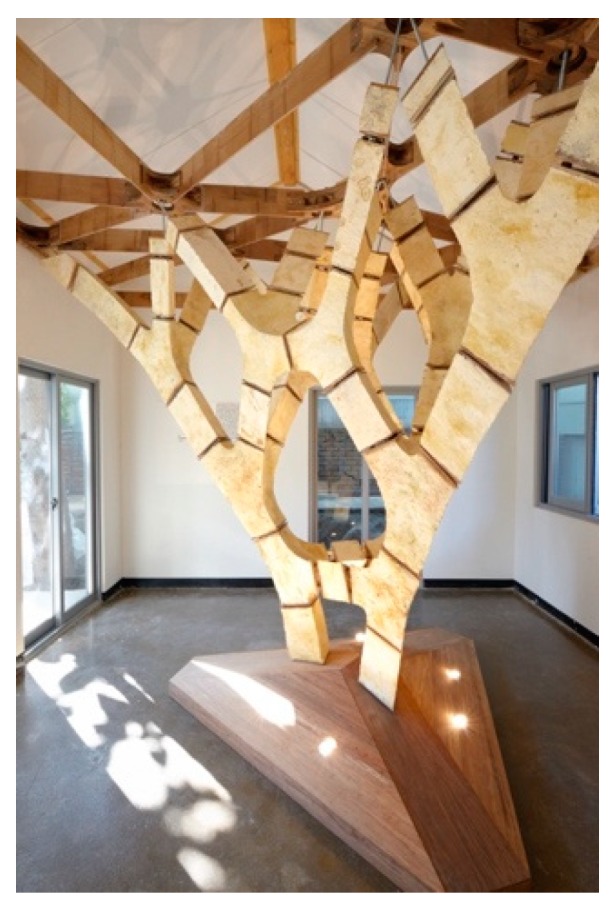
The MycoTree at the Seoul Biennale of Architecture and Urbanism, 2017 © Carlina Teteris.

In January 2017, an invitation to participate at the Seoul Biennale of Architecture and Urbanism in Korea offered the chance to produce a large enough, free-standing structure within an enclosed space. These parameters provided the basis for a prototypology named MycoTree (Figure 2), concentrating on the load-bearing capacities of mycelium-bound building elements while leaving out other questions such as water resistance or longevity [10]. The quickly formed interdisciplinary, intercontinental consortium of KIT, FCL, and the Block Research Group (BRG) of Swiss Federal Institute of Technology (ETH) in Zurich allowed for a very concentrated approach towards the questions how to calculate, form, and produce a spatial branching structure made out of load-bearing mycelium components, which was then produced in collaboration with Indonesian company Mycotech.

**Figure 3:**
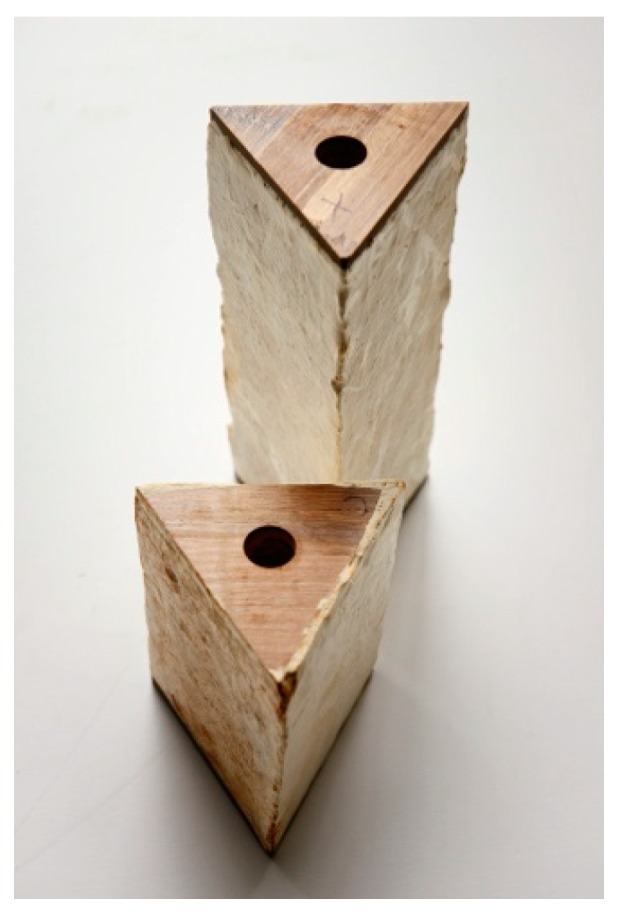
Building elements of MycoTree at FCL, 2017 © Carlina Teteris.

**Figure 4:**
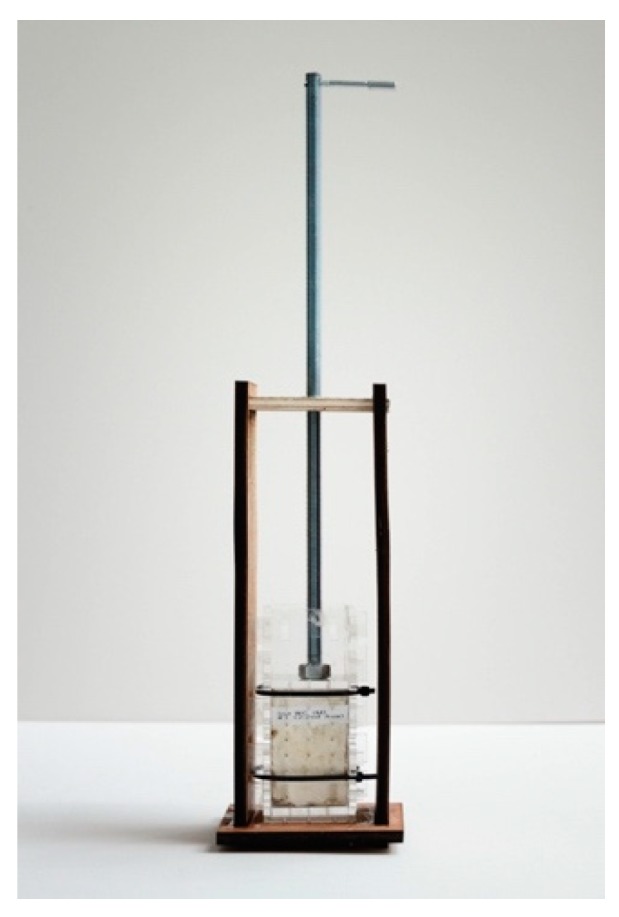
New production tools and methods for improved mycelium growth at FCL, 2017 © Carlina Teteris.

Up to this moment, mycelium components had never been used as structural and load-bearing elements, but only as self-supporting pieces in otherwise structured settings or art pieces. The aim to structurally activate the material however resulted in the reinvention of the growth recipe, the upscaling of such a process from the laboratory to a semi-industrial scale and the development of all necessary tools, as well as the engineering of a structural concept reacting to the material specifications of mycelium. MycoTree’s geometry was designed using 3D graphic statics [11], a novel method developed by the BRG that extends the traditional two-dimensional structural design technique to fully spatial systems. Funicular geometry has the advantage that stresses in it are very low. Achieving stability through geometry, rather than through material strength, opens up the possibility of using such weak materials as mycelium. Next to those technological developments, the concentrated work following the prototypological approach required as well an analysis of economic, ecologic, ethical, and psychological parameters of production and use of this new material and construction method (Figure 3 and Figure 4), responding to the expanded view of architectural research Till describes in his treatise.

**Figure 5:**
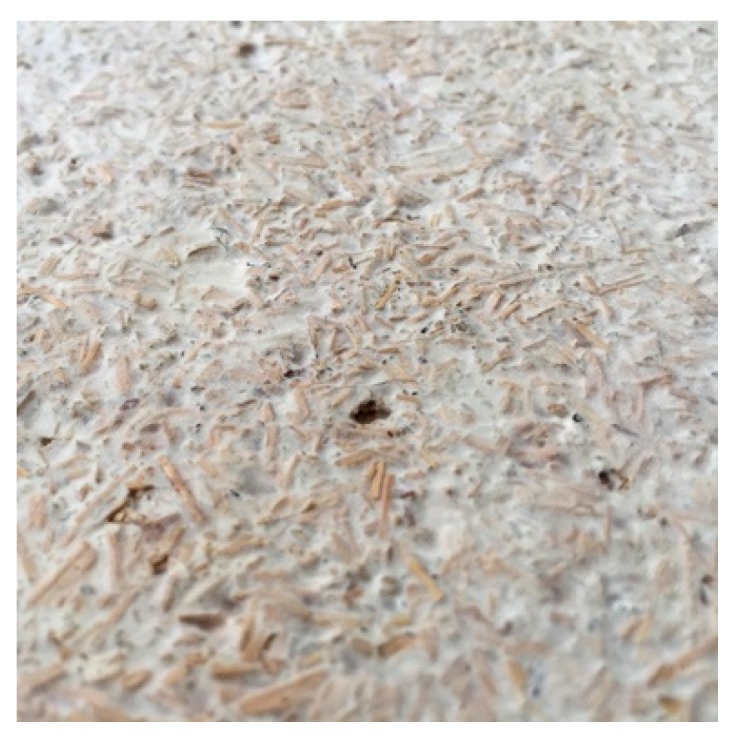
Close up image of mycelium insulation board at UMAR, 2018 © Felix Heisel.

**Figure 6:**
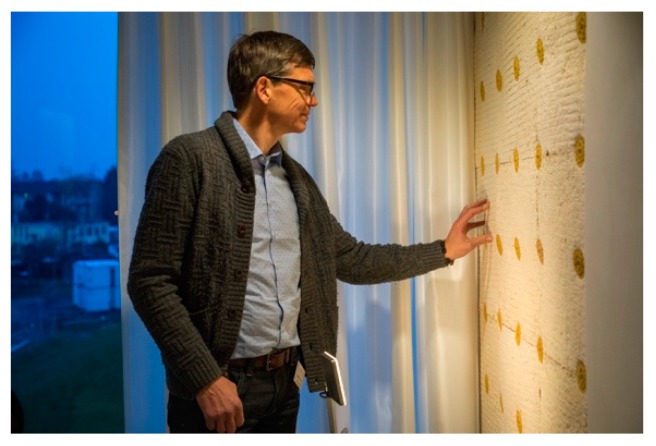
Wall elements as insulation and plaster base at UMAR, 2018 © Empa.

Following this first application, the research continued by incorporating mycelium elements within the prototypology UMAR (Urban Mining and Recycling) [12], a Swiss housing unit designed by Werner Sobek with Dirk Hebel and Felix Heisel at the Empa NEST in Dübendorf. Here, the material (produced in collaboration with American company Ecovative) was applied as an insulating wall cladding, serving at the same time as base for a loam plaster (Figure 5). Surface characteristics, robustness, and insulation qualities stood in the center of the investigation. Another aspect of the expanded field here became only obvious after the opening of the living unit: Visitors (Figure 6) started to question whether the mycelium material could be dangerous for the inhabitants—associating it with mold. The necessary communication and clarification of the fact that mycelium-bound building elements are dead organisms added another layer of complexity to the project: education.

**Figure 7:**
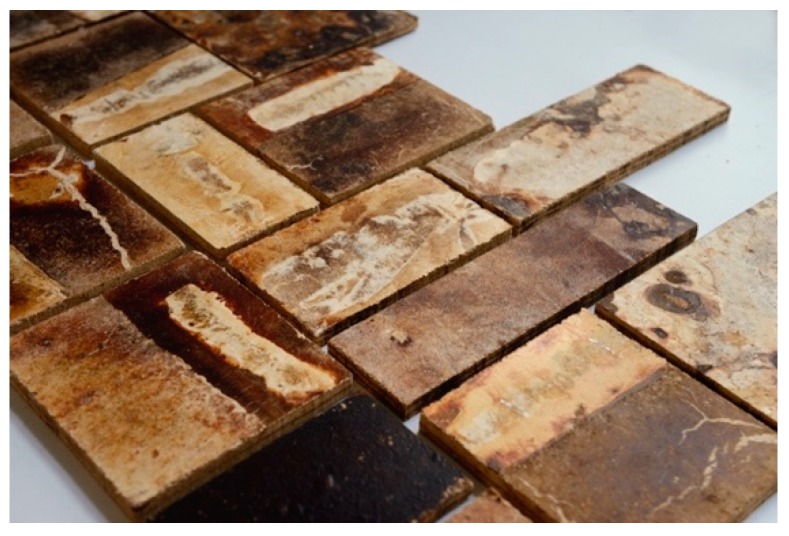
Construction panels produced out of mycelium at FCL, 2018 © Carlina Teteris.

**Figure 8:**
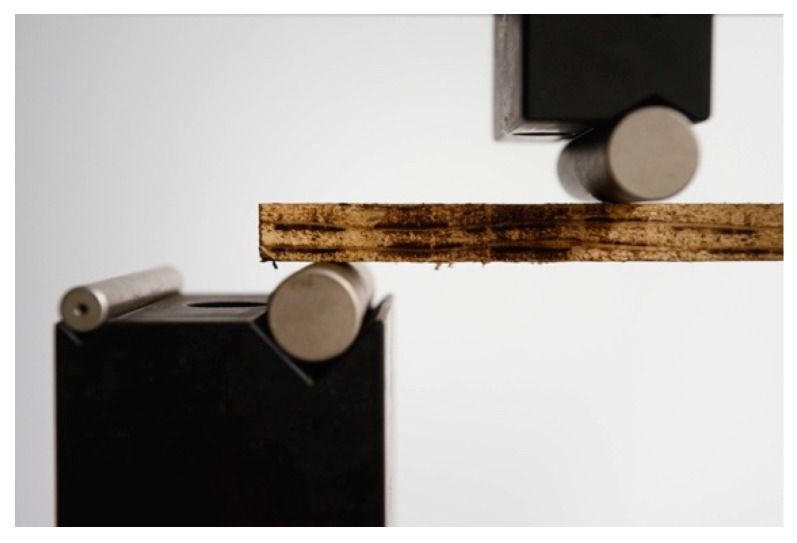
Bending test of mycelium boards at FCL, 2018 © Carlina Teteris.

As described in the first two prototypological applications, the question of longevity had been excluded from the investigations so far. A first application of mycelium-bound wall panels as an alternative to commonly used gypsum or particle boards was tested in a low-cost housing typology designed by Stephen Cairns and his FCL team on the island of Batam in Indonesia in 2018/2019 [13]. For this application, the team under the guidance of Dirk Hebel, Nazanin Saeidi, and Alireza Javadian developed a method of compacting mycelium materials in such a way that a tough and dense board (Figure 7 and Figure 8) could be manufactured—again in collaboration with company Mycotech. Through compacting, it was also possible to control the water uptake of the panels, thus protecting the panels from biological attacks. At the same time, the mechanical properties could be increased dramatically, which led to the next step of the development of the expanded body of the architectural research project.

**Figure 9:**
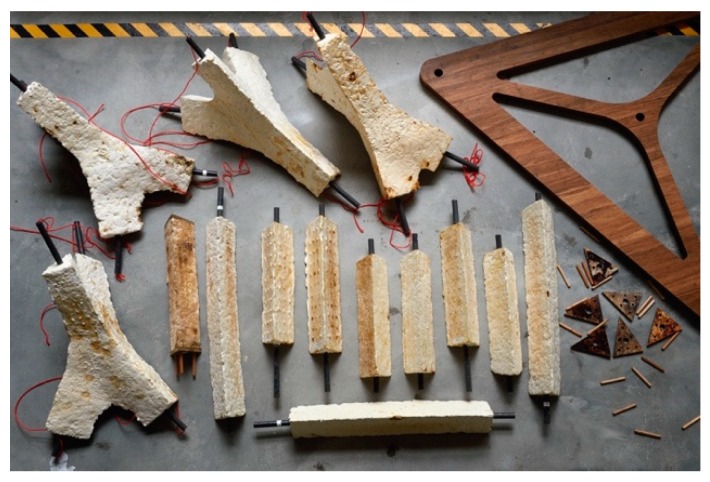
Set of parts for the prototypology Futurium Berlin, 2019 © Carlina Teteris.

Learning from the results of this series of prototypologies, KIT, ETH, and FCL produced another load-bearing structure for the Futurium in Berlin in 2019. Here, it was possible to increase the compression strength of the single mycelium-bound building elements significantly in comparison to the MycoTree prototypology from Seoul. This allowed for a slimmer and more elegant structure utilizing varying densities and strengths of the material depending on their respective location within the structure, and thus depending on their structural loads and necessities (Figure 9).

## 5. Conclusions

The methodology of a prototypological development allows for an understanding of architectural research not as “building a building”, but as building a continuous and self-informing body of knowledge within an expanded field. This field consists of physical applications as or within singular architectural prototypologies, as well as their representation and use, in the theories beyond the building, in the multiple interpretations of the building (Till), their social and cultural setting, and psychological framework. As described above, the single prototypology therefore acts as one element within a bigger, systematic research question and sometimes with a limited set of requirements. The advantage of such an approach lies in the adaptability of the prototypology within each new setting, allowing for the discovery of new opportunities unknown before. The article described and showcased this methodology with the help of a research project on innovative mycelium-bound building materials. Instead of showing an isolated and singular setting as a building, the article tried to describe the informative and open character of a continuous process, whereby the field of application is not determined in the beginning but can and will be found during the act of research and building.
